# Supplementary material for: No bejel among Surinamese, Antillean and Dutch syphilis diagnosed patients in Amsterdam between 2006–2018 evidenced by multi-locus sequence typing of Treponema pallidum isolates
Source: PLoS One. 2020 Mar 11;15(3):e0230288. doi: 10.1371/journal.pone.0230288 (PMC7065763; doi:10.1371/journal.pone.0230288)
Supplement: S1 Table — S1a) Table. Pearson Chi-squared test for ethnicity and typability of samples. S1b) Table. Fisher’s Exact test for RPR titer and typability of samples. S1c) Table. Pearson Chi-squared test for syphilis stage and typability of samples. S1d) Table. Fisher’s Exact test for HIV status and typability of samples. (DOCX) [file pone.0230288.s001.docx]

**S1 Table.** **Fisher’s Exact and Pearson Chi-squared Tests for ethnicity, RPR titer, syphilis stage and HIV status versus typability of the samples.** S1a) Table. Pearson Chi-squared test for ethnicity and typability of samples. S1b) Table. Fisher’s Exact test for RPR titer and typability of samples. S1c) Table. Pearson Chi-squared test for syphilis stage and typability of samples. S1d) Table. Fisher’s Exact test for HIV status and typability of samples.

A)

|  |  | Fully typed |  | Total |
| --- | --- | --- | --- | --- |
| Ethnicity |  | No | Yes |  |
|  | Antillean | 8 | 16 | 24 |
|  | Dutch | 17 | 50 | 67 |
|  | Surinamese | 9 | 37 | 46 |
| Total |  | 34 | 103 | 137 |
|  | *Pearson Chi-Square* | *p = 0.444* |  |  |

B)

|  |  | Fully typed |  | Total |
| --- | --- | --- | --- | --- |
| VDRL titer |  | No | Yes |  |
|  | high | 9 | 34 | 43 |
|  | low | 5 | 21 | 26 |
|  | middle | 12 | 30 | 42 |
|  | negative | 7 | 17 | 24 |
|  | unknown | 1 | 1 | 2 |
| Total |  | 34 | 103 | 137 |
|  | *Fisher's Exact Test* | *p = 0.651* |  |  |

C)

|  |  | Fully typed |  | Total |
| --- | --- | --- | --- | --- |
| Syphilis stage | | No | Yes |  |
|  | Primary stage | 22 | 86 | 108 |
|  | Secondary stage | 12 | 17 | 29 |
| Total |  | 34 | 103 | 137 |
|  | *Pearson Chi-Square* | *p = 0.02* |  |  |
|  |  |  |  |  |

D)

|  |  | Fully typed |  | Total |
| --- | --- | --- | --- | --- |
| HIV status |  | No | Yes |  |
|  | negative | 16 | 59 | 75 |
|  | positive | 16 | 40 | 56 |
|  | unknown | 2 | 4 | 6 |
| Total |  | 34 | 103 | 137 |
|  | *Fisher's Exact Test* | *p = 0.572* |  |  |
