# Supplementary material for: No bejel among Surinamese, Antillean and Dutch syphilis diagnosed patients in Amsterdam between 2006–2018 evidenced by multi-locus sequence typing of Treponema pallidum isolates
Source: PLoS One. 2020 Mar 11;15(3):e0230288. doi: 10.1371/journal.pone.0230288 (PMC7065763; doi:10.1371/journal.pone.0230288)
Supplement: S2 Table — (DOCX) [file pone.0230288.s002.docx]

**S2 Table.** **Fisher’s Exact test for syphilis stage versus genetic *Treponema pallidum* subspecies *pallidum* clade.**

|  |  | TPA genetic clade | |  | Total |
| --- | --- | --- | --- | --- | --- |
| Syphilis stage | | Unknown | Nichols-like | SS14-like |  |
|  | Primary stage | 22 | 18 | 68 | 108 |
|  | Secondary stage | 12 | 0 | 17 | 29 |
| Total |  | 34 | 18 | 85 | 137 |
|  | *Fisher's Exact Test* | *p = 0.006* |  |  |  |
