# Supplementary material for: No bejel among Surinamese, Antillean and Dutch syphilis diagnosed patients in Amsterdam between 2006–2018 evidenced by multi-locus sequence typing of Treponema pallidum isolates
Source: PLoS One. 2020 Mar 11;15(3):e0230288. doi: 10.1371/journal.pone.0230288 (PMC7065763; doi:10.1371/journal.pone.0230288)
Supplement: S3 Table — (DOCX) [file pone.0230288.s003.docx]

**S3 Table.** **Overview of full MLST *Treponema pallidum* subspecies *pallidum* types found in the public database [13].**

|  | **Country** |  |  |  |  |  |  | **Total** |
| --- | --- | --- | --- | --- | --- | --- | --- | --- |
| **type** | Australia | Cuba | Czech Republic | France | Portugal | Switzerland | The Netherlands |  |
| 1.1.1 | 0 | 1 | 19 | 18 | 7 | 9 | 20 | **74** |
| 1.1.10 | 0 | 1 | 0 | 0 | 0 | 0 | 3 | **4** |
| 1.1.11 | 0 | 0 | 0 | 1 | 0 | 0 | 0 | **1** |
| 1.1.13 | 0 | 0 | 0 | 1 | 0 | 0 | 0 | **1** |
| 1.1.16 | 0 | 0 | 1 | 0 | 0 | 0 | 0 | **1** |
| 1.1.3 | 0 | 0 | 5 | 0 | 0 | 2 | 0 | **7** |
| 1.1.8 | 0 | 0 | 31 | 5 | 0 | 0 | 4 | **40** |
| 1.1.9 | 0 | 0 | 0 | 1 | 0 | 0 | 0 | **1** |
| 1.11.8 | 0 | 0 | 0 | 3 | 0 | 0 | 0 | **3** |
| 1.17.9 | 0 | 0 | 0 | 2 | 0 | 0 | 2 | **4** |
| 1.18.1 | 0 | 0 | 0 | 1 | 0 | 0 | 0 | **1** |
| 1.19.1 | 0 | 0 | 0 | 1 | 0 | 0 | 0 | **1** |
| 1.22.12 | 0 | 0 | 0 | 1 | 0 | 0 | 0 | **1** |
| 1.23.1 | 0 | 0 | 0 | 2 | 0 | 0 | 0 | **2** |
| 1.26.1 | 0 | 0 | 17 | 0 | 0 | 0 | 0 | **17** |
| 1.28.1 | 0 | 0 | 1 | 0 | 0 | 0 | 0 | **1** |
| 1.29.1 | 0 | 0 | 1 | 0 | 0 | 0 | 0 | **1** |
| 1.3.1 | 0 | 66 | 60 | 80 | 13 | 17 | 43 | **279** |
| 1.3.3 | 0 | 0 | 0 | 0 | 0 | 0 | 1 | **1** |
| 1.3.5 | 0 | 0 | 0 | 0 | 0 | 1 | 0 | **1** |
| 1.3.7 | 0 | 0 | 0 | 0 | 0 | 1 | 0 | **1** |
| 1.31.1 | 0 | 0 | 1 | 0 | 0 | 0 | 0 | **1** |
| 1.32.1 | 0 | 0 | 1 | 0 | 0 | 0 | 0 | **1** |
| 1.32.10 | 0 | 0 | 0 | 0 | 0 | 0 | 1 | **1** |
| 1.36.1 | 0 | 0 | 4 | 0 | 0 | 0 | 0 | **4** |
| 1.4.1 | 0 | 0 | 1 | 0 | 0 | 3 | 0 | **4** |
| 1.43.1 | 0 | 0 | 0 | 0 | 0 | 0 | 2 | **2** |
| 1.44.1 | 0 | 0 | 0 | 0 | 0 | 0 | 2 | **2** |
| 1.46.3 | 0 | 0 | 0 | 0 | 0 | 0 | 1 | **1** |
| 1.47.1 | 0 | 0 | 0 | 0 | 0 | 0 | 1 | **1** |
| 1.5.1 | 0 | 0 | 0 | 0 | 0 | 2 | 0 | **2** |
| 1.8.1 | 0 | 0 | 0 | 0 | 0 | 1 | 0 | **1** |
| 1.9.1 | 0 | 0 | 0 | 0 | 0 | 1 | 0 | **1** |
| 13.1.1 | 0 | 0 | 0 | 0 | 0 | 1 | 0 | **1** |
| 14.3.1 | 0 | 0 | 0 | 1 | 0 | 0 | 0 | **1** |
| 15.7.3 | 0 | 5 | 0 | 0 | 0 | 0 | 0 | **5** |
| 16.3.1 | 0 | 1 | 0 | 0 | 0 | 0 | 0 | **1** |
| 17.1.1 | 0 | 0 | 1 | 0 | 0 | 0 | 0 | **1** |
| 18.1.1 | 0 | 0 | 1 | 0 | 0 | 0 | 0 | **1** |
| 19.3.1 | 0 | 0 | 0 | 0 | 0 | 0 | 4 | **4** |
| 2.1.2 | 0 | 0 | 0 | 1 | 0 | 2 | 0 | **3** |
| 20.2.10 | 0 | 0 | 0 | 0 | 0 | 0 | 1 | **1** |
| 3.2.3 | 0 | 0 | 0 | 1 | 0 | 2 | 11 | **14** |
| 4.1.1 | 0 | 0 | 1 | 0 | 0 | 0 | 0 | **1** |
| 4.3.1 | 0 | 0 | 0 | 1 | 0 | 0 | 0 | **1** |
| 5.3.8 | 0 | 0 | 0 | 1 | 0 | 0 | 0 | **1** |
| 6.3.1 | 0 | 0 | 0 | 1 | 0 | 0 | 0 | **1** |
| 7.1.9 | 0 | 0 | 0 | 0 | 5 | 0 | 0 | **5** |
| 7.45.9 | 0 | 0 | 0 | 0 | 0 | 0 | 1 | **1** |
| 9.14.3 | 1 | 0 | 0 | 0 | 0 | 0 | 0 | **1** |
| 9.2.3 | 0 | 0 | 0 | 0 | 0 | 0 | 2 | **2** |
| 9.20.3 | 0 | 0 | 0 | 2 | 0 | 0 | 0 | **2** |
| 9.24.8 | 0 | 2 | 0 | 0 | 0 | 0 | 0 | **2** |
| 9.25.3 | 0 | 1 | 0 | 0 | 0 | 0 | 0 | **1** |
| 9.7.3 | 0 | 0 | 3 | 7 | 0 | 0 | 4 | **14** |
| **Total** | **1** | **77** | **148** | **131** | **25** | **42** | **103** | **527** |
